# Slow motion in films and video clips: Music influences perceived duration and emotion, autonomic physiological activation and pupillary responses

**DOI:** 10.1371/journal.pone.0199161

**Published:** 2018-06-22

**Authors:** Clemens Wöllner, David Hammerschmidt, Henning Albrecht

**Affiliations:** Universität Hamburg, Hamburg, Germany; University of Zurich, SWITZERLAND

## Abstract

Slow motion scenes are ubiquitous in screen-based audiovisual media and are typically accompanied by emotional music. The strong effects of slow motion on observers are hypothetically related to heightened emotional states in which time seems to pass more slowly. These states are simulated in films and video clips, and seem to resemble such experiences in daily life. The current study investigated time perception and emotional response to media clips containing decelerated human motion, with or without music using psychometric and psychophysiological testing methods. Participants were presented with slow-motion scenes taken from commercial films, ballet and sports footage, as well as the same scenes converted to real-time. Results reveal that slow-motion scenes, compared to adapted real-time scenes, led to systematic underestimations of duration, lower perceived arousal but higher valence, lower respiration rates and smaller pupillary diameters. The presence of music compared to visual-only presentations strongly affected results in terms of higher accuracy in duration estimates, higher perceived arousal and valence, higher physiological activation and larger pupillary diameters, indicating higher arousal. Video genre affected responses in addition. These findings suggest that perceiving slow motion is not related to states of high arousal, but rather affects cognitive dimensions of perceived time and valence. Music influences these experiences profoundly, thus strengthening the impact of stretched time in audiovisual media.

## Introduction

The award-winning movie “Forrest Gump” [1] contains a remarkable scene in which Forrest, a boy with impaired motion, is chased by bullying children on their bikes. He starts running, shaking off his leg braces to escape the bullies. This is a significant turning point in his life, as Forrest becomes a very fast runner and eventually a top athlete. This film scene is produced in slow motion, allowing the film viewer to perceive facial expressions and the surroundings in a greater amount of detail. The slowness may also allow for contemplation of the emotional transition from threat to overcoming and success. The highly emotive film music emphasises the situation in the scene, and the running in slow motion may seem to be even faster in internally assumed time. Slow-motion scenes such as the one described above are very common across a range of media genres, yet there is only sparse empirical research examining why they are used and how they may possibly relate to daily-life experiences. The aim of the current study was to investigate the emotional effects of slow-motion footage on viewers. Drawing on psychological theories of time distortion in heightened situations, we assumed that stretched time in screen-based media would have an impact on perceived emotion, physiological responses and visual perception. Music typically plays a key role in these experiences, so we contrasted slow-motion and real-time motion scenes in an experiment using multimodal conditions with and without music.

### Time perception and time distortions

Since the early days of film-making, producers, directors and projectionists experimented with stretching or accelerating time by varying shooting and playback speed—cameras and projectors were operated by turning a handle. “Time lapse” or the acceleration of time for processes involving objects and larger scenes such as the growing and blossoming of a flower, cloud fluctuations in the sky or the traffic circulation in a city offers the viewer an impression of processes that otherwise extend beyond the capacity for temporal integration in human memory systems [2],[3]. Yet accelerating human motion in film often leads to amusing effects. Slowing down, on the other hand, makes details of very fast processes such as explosions visible, a phenomenon known as “bullet time” in film-making. Moreover, slow-motion scenes offer new aesthetic experiences when presenting the fine-grained movement details of skilled athletes or dancers. Even more, the stretched time in these scenes seem to mirror inner processes of heightened emotional states and may invoke such experiences for the viewer.

Time perception varies impressively, and it is a long-established observation in philosophy and psychological research that the outer circumstances as well as the inner state of a person may lead to experiences of time that seems to stand still or to fly [4],[5]. Particularly in life-threatening situations, people may perceive time to be slowed down to extreme extents. In a survey of people who had experienced such situations, including climbers who fell, Noyes and Kletti [6] reported that three quarters of individuals experienced distortions of time. In addition to time being slowed down, they also frequently reported a higher alertness and speed in cognitive functioning. This enabled them to perceive numerous details that they would not have noticed otherwise. Arstila [7] proposes that individuals have a sense of normal speed in which they perceive events and act in the world. In frightening situations, this sense is distorted by a mechanism that, partially substantiated by higher activation of the norepinephrine system, increases arousal and heightens perception and attention. These additional resources enhance and speed up cognitive processes. As a result, individuals are under the impression of having more time for perceiving events and for responding. Internal comparisons with the sense of normal speed then give rise to the experience of time as being slowed down, and individuals tend to overestimate durations in these situations.

Music has a profound impact on time perception [8], [9]. In “Principles of Psychology”, James ([5], p. 618) already observed that “tracts of time filled (with clicks of sound) seem longer than vacant ones of the same duration” (parenthesis in the original), suggesting that subdivisions of time as they occur in music provide more information that lead to longer time estimates. Characteristics of the music such as tempo should thus have an impact on time perception. In a study individuals were asked to queue for a service while background music was played [10]. The music was either slowed down (minimum tempo: 109 bpm) or sped up (maximum 179 bpm). Faster versions increased perceived duration, so that participants overestimated their waiting time. The slow-music condition led to underestimations of time.

Other factors such as the music’s arousal, valence or familiarity may further influence time perception. In an experimental study, Droit-Volet and colleagues [11] manipulated the tempo of musical excerpts by digitally speeding them up. The faster versions, containing a higher number of events in a given time span, were not only judged to be more arousing, they also led to overestimations of duration. The emotional quality or valence of the music, for instance as being happy versus sad (or pleasant versus unpleasant), also influenced findings in that pleasant music led to shorter duration judgments. Cameron and colleagues [12] found that the more individuals like the music, the shorter they experience wait length. This result is corroborated for familiar music that led to shorter estimates of time intervals, but only if participants were waiting “idly” without solving a secondary task [13], while secondary tasks influence cognitive load and subsequent duration estimates [14].

### Emotional and pupillary responses induced by music

Music is a powerful means of inducing arousal and emotional valence that may in turn influence time perception. Over the past years, an increasing number of studies have investigated psychophysiological responses to emotional music. Most researchers agree that music can indeed *induce* emotions rather than only convey emotional states that people may recognise ([15], [16], for a review, see [17]). Among the physiological responses measured most frequently are Galvanic skin response (GSR), indicating higher electrodermal conductance due to increased skin moisture when individuals are activated or aroused; as well as respiration rate (RSP) and heart rate (HR), both of which typically also increase in states of higher arousal [18].

Emotions induced by music bear similarities with responses to further real-life events and inductions by other sensory stimuli. Strong emotional responses that may be experienced as “chills” by participants were recorded for musical excerpts, non-musical sounds, tactile, gustatory and visual sensory information [19]. Emotional responses are particularly strong for film music [20]. Ellis and Simons [21] paired short visual film excerpts (lasting six seconds) with instrumental music that was not originally intended for the film scenes. Film excerpts that were high in arousal resulted in larger GSR, and HR slowed down in excerpts with negative valence. Music had an additive effect on subjective ratings of the films’ valence and arousal, meaning that perceptions of the film excerpts were stronger in these dimensions when music was present. The influence of music on physiological responses, on the other hand, was more complex. Increases in GSR were observed for highly arousing music, but only for films with positive valence. These findings are in line with an earlier study [22] which showed that GSR increased with highly arousing music. More recently, White and Rickard [23] presented emotional film music excerpts to participants and found that GSR and HR both declined, suggesting a relaxing effect of the music, independently of whether the music had been rated to be happy or sad. Increases in GSR were observed for happy music [15] and decreases for sad music, and RSP was higher with fast music [24]. The combined presentation of music with visual stimuli such as emotional pictures leads to an increase in GSR, RSP, and HR [25], as well as brain structures associated with emotional processing [26].

Music has been shown to be effective in inducing emotions that may in turn affect visual attention and physiological responses of the eye including eye blinks and pupillary responses. While an earlier study [27] observed startle-reflex eye blinks even for low-intensity sounds, more recent research [16] found that unpleasant music elicited larger blink amplitudes with a smaller latency. Pleasant music, on the other hand, increased GSR to a higher extent. In a study using an attentional blink task, in which participants had to ignore numbers but identify letters that were shown in rapid succession, they performed best in a sad emotion condition, in which the music induced low arousal and negative valence [28]. Bradley and colleagues [29] combined measures of autonomic activity (mean GSR and HR changes) with pupillary responses in an experiment involving picture viewing. Emotional pictures, as compared to neutral ones, led to increases in pupil size and GSR. Heart rate was only decelerated for negative valence (unpleasant pictures). The finding that changes in GSR were related to changes in pupillary diameter suggests a common underlying arousal mechanism for both responses. In another study using musical stimuli [30], individuals’ subjectively felt arousal and tension were correlated with pupillary responses, providing further evidence for arousal induction by music that affect the diameter of the pupil (see also [31]).

Further research using eye-tracking found that viewers attend differently to details in images depending on their music-induced emotions [32, 33]. When watching visual stimuli with music or in silence in an experimental study, the music reduced eye movements in terms of longer fixations, a higher number of blinks and fewer saccades, arguably related to a shift of attention to an “inner world” ([34], p. 556). Music may thus induce states of contemplation and attention to inner processes. In addition, music can aid in memorisation and recognition of visual film material [35], and guide attention as measured by eye movements [36].

### Aims and hypotheses

Taken together, film directors have often used slow motion (SloMo) for creating momentous emotional scenes [3], [37], [38]. Music may increase the physiological dimension of emotional responses to such film scenes, particularly with regard to arousal. The psychometrical and psychophysiological functioning of SloMo as a central topic of our research has not been analysed in detail, and, to our knowledge, no research has investigated the effects of SloMo in audiovisual media on the oculomotor system or peripheral physiology. We aim at elucidating some of the psychophysiological underpinnings for the success of highly popular SloMo footage in films and on streaming channels (cf. [39]). Such SloMo scenes typically present sports and dance actions combined with highly emotional music.

The current study investigated slow-motion film excerpts compared to the same excerpts in adapted real-time motion. We analysed the influence of SloMo and music on duration estimates, perceived emotions as well as pupillary responses and autonomic activity as indicators of arousal. A particular focus was on interactions between auditory and visual information. First, we hypothesised that SloMo has an impact on perceived emotion (arousal, valence), induced emotion in terms of arousal (peripheral physiological activation, pupillary responses) across different genres of video excerpts. Second, we assumed that music modulates these processes to a significant extent.

## Methods

### Participants

A total of forty-six participants (mean age: 23.59, *SD* = 4.31 years), 25 of them male, took part in the study. They all had normal hearing, and all but one had normal or corrected-to-normal vision. Their musical experience varied from no experience in playing a musical instrument up to 15 years of instrumental lessons (median: 8.0 years). They rated their own active experiences (from 1 “not at all” to 7 “very much”) in film making at *M* = 2.37 (*SD* = 1.66), in sports at *M* = 2.91 (*SD* = 1.33) and in dance or ballet at *M* = 1.98 (*SD* = 1.66). Thus participants were generally not very experienced in any of these genres. As specified below, the number of participants was reduced for some analyses due to outliers or occasional equipment failure. This study was approved by the Ethics Committee at the Faculty of Humanities (EGKW), Universität Hamburg, and all participants provided informed consent.

### Design and stimulus material

In a multimodal repeated-measures design, participants watched video excerpts of commercial films, dance and sports footage. Videos were presented either in original slow motion or in adapted real-time motion. Videos were shown with music (audiovisual: AV) and without music (visual-only: V). Hence there were three main factors: Tempo (SloMo, real-time motion), Modality (AV, V), and Genre (film, ballet, sports).

For each genre, three excerpts were chosen that contained a SloMo scene for at least 15 seconds. These video excerpts stemmed from popular film or video clips that were commercially available or found on a popular streaming channel in sufficient resolution, and were chosen under the preconditions that a) human movement was slowed down (i.e., SloMo of physical objects such as in “bullet time” was not considered), and b) no tempo alterations were made within the excerpt in terms of varying playback speed. The durations of the original SloMo excerpts ranged from 16–40s (Table 1).

**Table 1:** Details of the nine SloMo and real-time excerpts used in the current study.

Excerpts		Start of excerpt	Duration (s)		Acceleration quotient
			Original SloMo	Adapted real-time motion	
**Films**	Forrest Gump [1]	00:16:32	26.20	10.92	2.4
	A Clockwork Orange [40]	00:31:56	22.07	5.66	3.9
	Silent Youth [41]	00:69:00	40.00	20.00	2.0
**Ballet**	Vienna SloMo Ballet [42]	00:01:33	16.08	4.34	3.7
	Ballet Adagio [43]	00:04:42	35.00	11.65	3.0
	Slow Motion Ballet [44]	00:00:41	39.72	11.34	3.5
**Sports**	Badminton [45]	00:00:27	30.16	2.75	11.0
	Table Tennis [46]	00:01:09	21.40	2.67	8.0
	Roger Federer [47]	00:03:05	36.25	3.60	10.0

In a pilot study, the speed rates of the videos were adjusted (using *Adobe Premiere Pro CS6*) so as to match real-time movements of humans. Four researchers, among them the three authors, rated the adapted speed rates of each excerpt, and further tempo adjustments were made until it was unanimously agreed that real-time motion scenes matched natural human movements. The factor by which the original footage had been slowed down ranged from 2 (Silent Youth) to 11 (Badminton), with higher factors for the three sports excerpts. There were only a few visual cuts in the original SloMo scenes, and the music was generally not aligned to cuts. In order to keep the duration of the music consistent across conditions, videos in the adapted real-time motion condition were presented three times with a black screen of 2s in between, while the same music, unaltered in tempo, was played as in the SloMo scenes.

### Procedure

Participants were presented with all conditions in individually randomised orders and counter-balanced blocks. They watched the stimuli on a 24″ computer screen (1920 x 1200 pixel resolution) at a distance of 60–80 cm. Stimuli covered 75% at the centre position of the screen with black edges on each side. The music was played via headphones (*Beyerdynamic DT-880 Pro*). After providing informed consent, the physiological recording device (*Nexus*, *MindMedia*) was attached to participants for measuring respiration rate (RSP), Galvanic skin response (GSR) and heart rate (HR, measured by changes in blood volume pressure). Subsequently, the eye tracker (REDn, *Sensomotoric Instruments*; sample frequency: 60 Hz) was calibrated.

All material and questions were presented to participants using the software E-Prime 2 Pro (*Psychology Software Tools*). In order to record baseline measures, they first watched a screen showing a “relaxing” marine landscape for 60s. In experimental trials, there was a white fixation cross at the centre before a new video excerpt appeared, to be followed by the video itself and questions to be answered. After each trial, participants pressed a key to continue the experiment.

After the baseline recording, participants were presented with a block comprising all ballet, all sports or all film excerpts, followed by the other two blocks of genres in counterbalanced order. Each block contained the SloMo and real-time versions of the excerpts in AV and V conditions. Within each block, stimuli were randomised individually per participant. Following each stimulus presentation, participants rated the perceived duration, arousal and valence of the material. Out of the three repeated presentations in real-time stimuli, participants estimated the duration only of the first presentation. The eye tracker was calibrated anew before each block. The whole experiment lasted between 50–70 minutes.

### Data analysis

Repeated-measures ANOVAs were run on duration estimates and emotion judgments, pupil diameter as well as physiological responses. If data did not meet the sphericity assumption, a Greenhouse-Geisser correction was used. Post-hoc comparisons were calculated with a Bonferroni adjustment. If individual data points were missing, or were more than three standard deviations beyond the mean of the sample (outliers), data from these participants were excluded list-wise for a given measure.

Individual duration estimates were divided by the actual clock-time duration and subtracted by 1; thus normalised deviations were analysed across stimuli. Data of one participant were removed because of a number of outliers, and due to missing data and equipment failure, the data of further participants could not be used. Consequently, data from *n* = 39 for duration estimates and *n* = 40 for perceived emotion judgments were entered into analyses. Physiological responses of GSR, RSP and HR were averaged per participant and stimulus. Following Bradley and colleagues [29], values of the baseline condition were subtracted from physiological response measures. Data were aggregated across stimuli for a given genre before subjected to ANOVA. Due to equipment failure or missing data, data from five participants had to be discarded, leading to *n* = 41 data sets analysed.

For the time series of pupillary responses, zero data were assumed to be caused by blinks and not included in analyses. Since the baseline condition included looking at a still image with different visual parameters compared to experimental stimuli, pupillary dilations were not subtracted from it. Pupil diameters were averaged over both eyes and normalised over time to reach one mean value per participant and stimulus. One participant was excluded because he had only one functional eye. The number of participants for which pupillometry data could be analysed was *n* = 39.

## Results

### Duration estimates and judgments of arousal and valence

**Fig 1:**
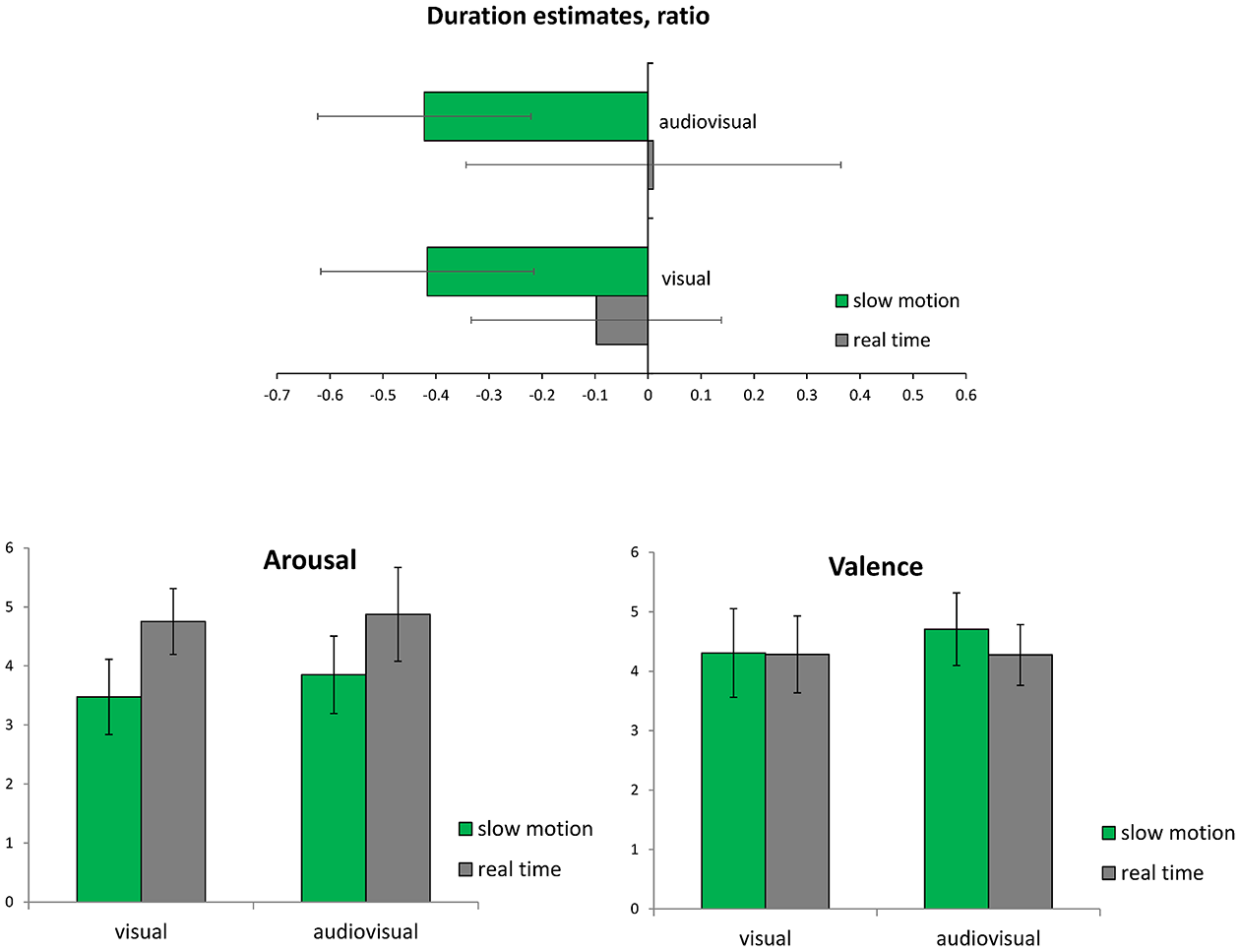
Duration estimates (top) and judgments of arousal and valence (lower left and right panels; *M*, *SD*). Duration estimates are normalised so that 0 indicates a perfect match to clock-time duration, and -1 half as long, +1 twice as long).

Duration estimates differed from clock-time in that stimuli were generally judged to last shorter (Fig 1, upper panel). There was a significant main effect for Tempo [*F*(1, 38) = 260.27, *p* < .001, *ƞ*_*P*_^2^ = .87], indicating that slow-motion video excerpts were underestimated to a larger extent than real-time motion videos. In other words, participants believed that the SloMo excerpts lasted shorter than they actually were, and underestimated the time more than for real-time excerpts. The second main factor Modality influenced results [*F*(1, 38) = 9.42, *p* < .01, *ƞ*_*P*_^2^ = .20], with audiovisual presentations leading to more accurate judgments as compared to visual-only presentations. Participants were also influenced by Genre in their duration estimates [*F*(1, 38) = 20.48, *p* < .001, *ƞ*_*P*_^2^ = .35]. Post-hoc analyses show that sport scenes were judged more accurately than ballet or film scenes (both *p* < .001). There were interaction effects for Tempo and Modality [*F*(1, 38) = 11.76, *p* < .01, *ƞ*_*P*_^2^ = .24], and Tempo and Genre [*F*(1.60, 60.63) = 27.70, *p* < .001, *ƞ*_*P*_^2^ = .42], which are related to the relatively precise duration estimates for real-time stimuli with music, particularly for sport excerpts.

Arousal was perceived to be higher in real-time excerpts as compared to SloMo excerpts [*F*(1, 39) = 188.12, *p* < .001, *ƞ*_*P*_^2^ = .83] (Fig 1, bottom left). There was a main effect for Modality with higher judgments for audiovisual compared to the visual presentations [*F*(1, 39) = 9.64, *p* < .01, *ƞ*_*P*_^2^ = .20]. Tempo and Modality interacted significantly [*F*(1, 39) = 5.62, *p* < .05, *ƞ*_*P*_^2^ = .13], indicating that the presence of music (AV) influenced perceived arousal particularly in the SloMo excerpts. There was also a main effect of Genre [*F*(2, 78) = 29.51, *p* < .001, *ƞ*_*P*_^2^ = .43], which in post-hoc analyses showed that both the sports and film excerpts were judged to be higher in arousal than the ballet excerpts (both *p* < .001). Genre interacted with the factors Tempo and Modality (both *p* < .001).

Valence was judged higher in SloMo [*F*(1, 39) = 10.65, *p* < .01, *ƞ*_*P*_^2^ = .21], and higher in AV presentations [*F*(1, 39) = 6.34, *p* < .05, *ƞ*_*P*_^2^ = .14] (Fig 1, bottom right panel). An interaction effect indicates that music increased perceived valence in SloMo, but not in real-time video clips [*F*(1, 39) = 11.96, *p* < .01, *ƞ*_*P*_^2^ = .24]. There was a main effect of Genre [*F*(2, 78) = 6.03, *p* < .01, *ƞ*_*P*_^2^ = .13], showing that ballet scenes were judged higher in valence as compared to films and sports excerpts (both *p* < .001). There were no interactions with Genre.

In summary, duration was systematically underestimated for SloMo excerpts and more precise for the shorter durations of real-time stimuli. Music enhanced the accuracy of duration estimates. While arousal was judged to be higher in conditions with music and for real-time excerpts, valence was higher in SloMo videos and in AV conditions. These results were further influenced by video genre.

### Physiological activation

**Fig 2:**
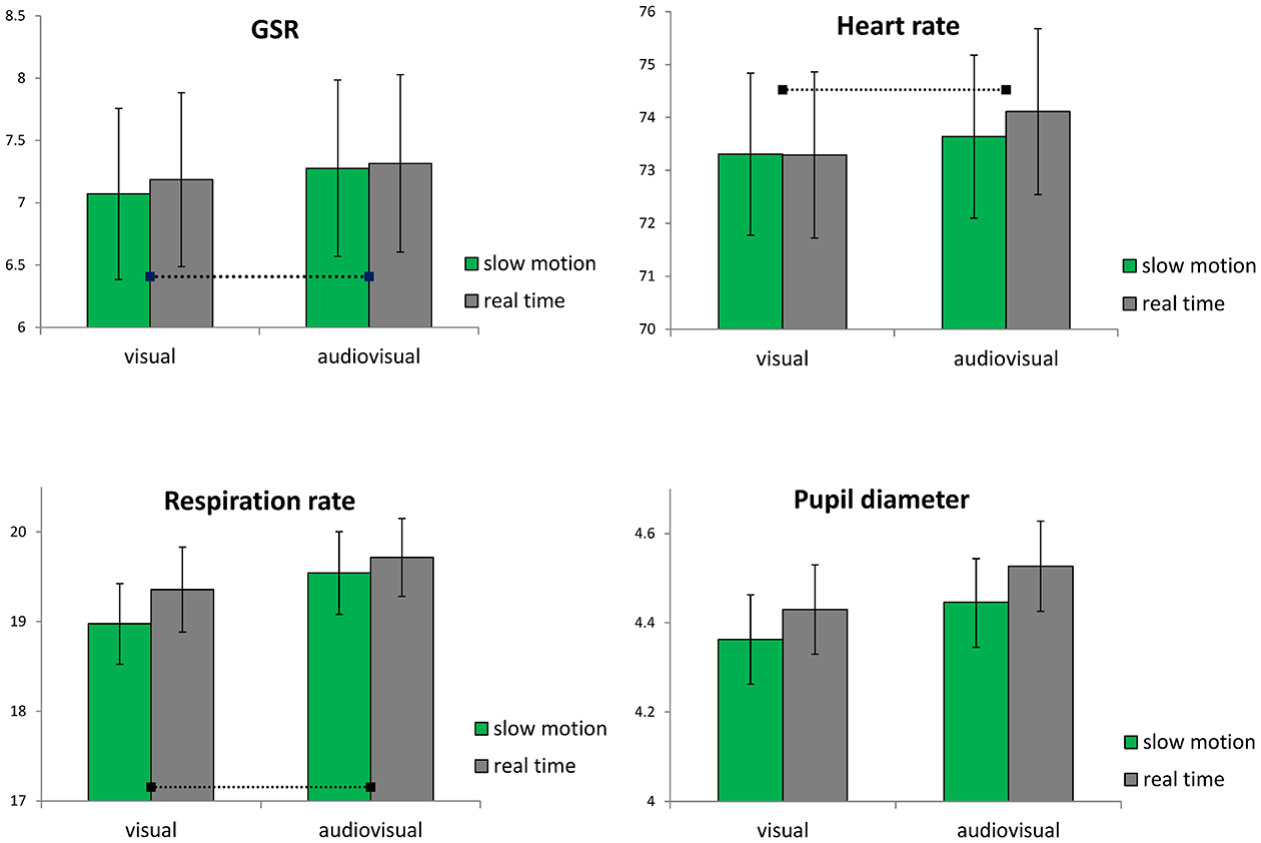
Physiological responses and pupil diameter (*M*, *SEM*). Dotted lines in results for GSR, HR, and RSP represent mean responses in the baseline condition, from which individual deviations were calculated and entered into statistical analyses.

Galvanic skin response (GSR) and respiration rate (RSP) were higher in experimental conditions than in the baseline measurement (still image), while heart rate (HR) was lower in experimental conditions (Fig 2). The following calculations are based on individual differences between values in the experimental and the baseline condition.

GSR did not differ according to Tempo [*F*(1, 40) = 2.01, *p* > .05, *ƞ*_*P*_^2^ = .05]. There was a significant main effect for Modality [*F*(1, 40) = 22.81, *p* < .001, *ƞ*_*P*_^2^ = .36], indicating that music increased GSR compared to no music (Fig 2, top left). While there were no overall differences between the three Genres [*F*(1.30, 51.91) = 1.06, *p* > .05, *ƞ*_*P*_^2^ = .03], there was a significant interaction between Genre and Tempo [*F*(2, 80) = 3.53, *p* < .05, *ƞ*_*P*_^2^ = .08], suggesting that for sports videos, tempo led to slightly higher skin conductance in real-time versions. This interpretation has to be treated with caution, since no main effects were observed for these two factors. There were no further interactions.

HR did not change over the two Tempo versions [*F*(1, 40) = 1.20, *p* > .05, *ƞ*_*P*_^2^ = .03]. There was a significant increase in HR according to Modality [*F*(1, 40) = 8.84, *p* < .01, *ƞ*_*P*_^2^ = .18], with higher mean HR in the audiovisual condition (Fig 2, top right). There was no effect for Genre [*F*(1.55, 61.98) = 1.24, *p* > .05, *ƞ*_*P*_^2^ = .03] and no interactions.

Analyses of RSP yielded a main effect of Tempo [*F*(1, 40) = 7.12, *p* < .05, *ƞ*_*P*_^2^ = .15], indicating that participants were breathing more often in the real-time versions (Fig 2, bottom left). There was also a significant main effect of Modality [*F*(1, 40) = 14.62, *p* < .001, *ƞ*_*P*_^2^ = .27], suggesting that music increased RSP. Genre did not cause differences in RSP [*F*(1.69, 67.47) = 2.31, *p* > .05, *ƞ*_*P*_^2^ = .06], and there were no interactions.

Taken together, there were significant effects for Modality, indicating that the presence of music led to higher arousal in terms of GSR, HR, and RSP changes, irrespective of the three video genres. RSP was the only physiological measure that was higher in adapted real-time motion compared to SloMo.

### Pupillary dilations

**Fig 3:**
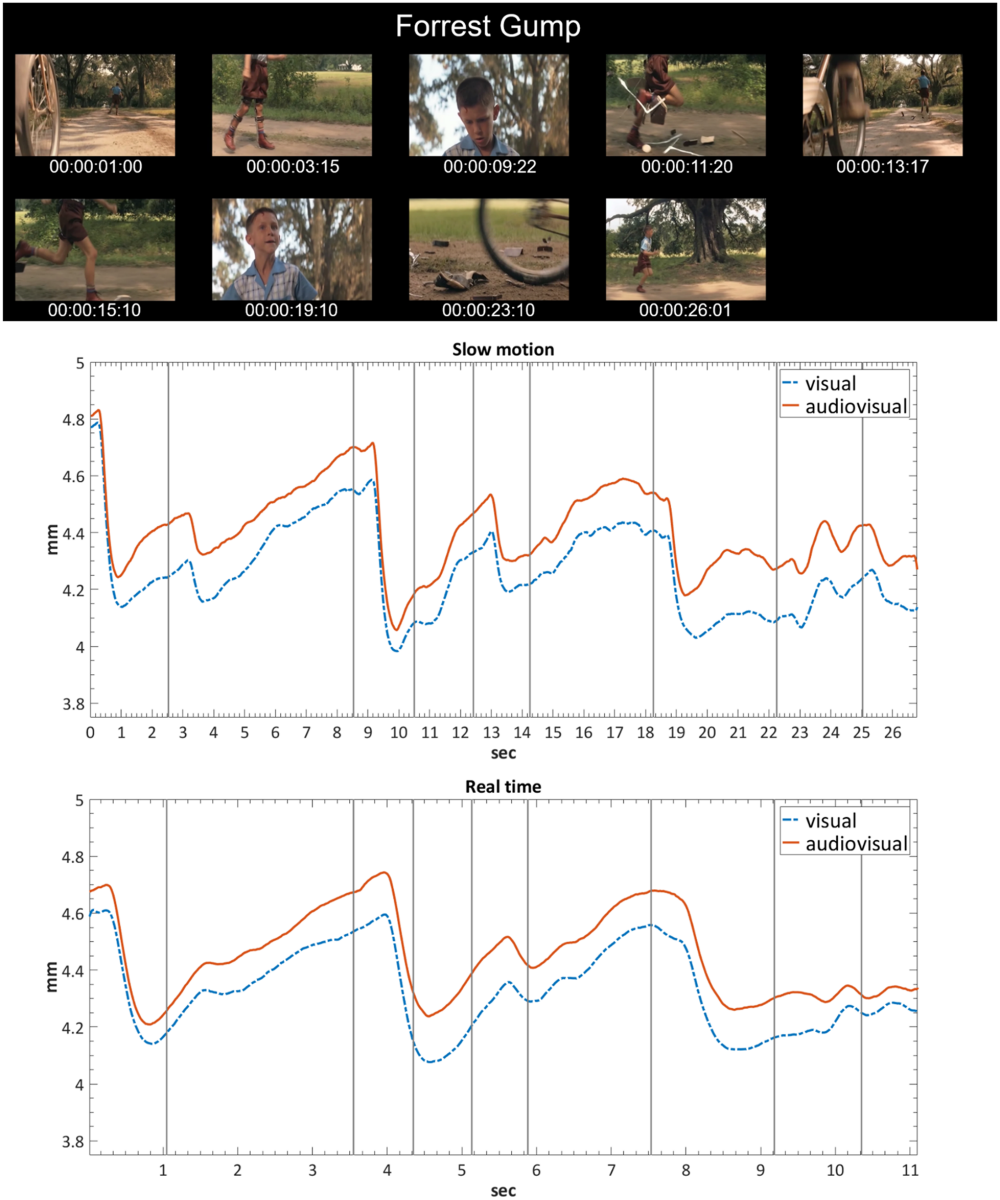
Stills from the film “Forrest Gump” and pupillary responses. Stills are taken approximately one second after shot cuts (top; time units are based on 25 fps timecode). Pupillary responses for the excerpt in SloMo (centre) and real-time motion (bottom). Time series were interpolated per participant in order to account for zero data caused by blinks, using the Piecewise Cubic Hermite Interpolating Polynomial function in Matlab (R2016b, *MathWorks*). Vertical lines represent shot cuts.

Changes in pupil diameter were recorded for all factors (Fig 2, bottom right). Averaged profiles show remarkable parallels across conditions (Fig 3). Tempo affected pupillary responses significantly [*F*(1, 38) = 19.12, *p* < .001, *ƞ*_*P*_^2^ = .33], with larger mean pupil diameters for adapted real-time videos, indicating higher levels of arousal when excerpts were shown in normal speed as compared to SloMo. There was also a significant main effect for Modality [*F*(1, 38) = 93.79, *p* < .001, *ƞ*_*P*_^2^ = .71], showing that the presence of music increased mean pupil diameter. As expected, participants’ pupil size changed according to Genre [*F*(2, 76) = 70.98, *p* < .001, *ƞ*_*P*_^2^ = .65], and post-hoc analyses indicate that dance scenes led to the largest pupil size, followed by sports and film scenes (all differences significant at *p* < .001). There was a significant interaction between Tempo and Genre [*F*(2, 76) = 4.47, *p* < .05, *ƞ*_*P*_^2^ = .11], suggesting influences of the specific visual material.

These results show that arousal as measured by pupil diameter was most strongly affected by the presence of music, but also by the differences in the visual material according to presentation tempo and genre.

## Discussion

Experiences of distorted time have often been reported for highly emotional situations. In this paper, we proposed that slow motion in audiovisual screen-based media mirrors the psychological processes involved in these situations, and that music modulates the observers’ experiences. Nine SloMo excerpts from commercial films, ballet and sports footage were shown with and without music. Corresponding accelerated versions were also presented that matched real-time human movements. SloMo videos were not only systematically underestimated in duration, they also led to lower perceived arousal as well as lower bodily arousal responses in terms of respiration rate and pupil diameters. Valence, on the other hand, was reported as higher in SloMo. Music facilitated more precise duration estimates, increased perceived arousal and valence, and also affected bodily responses with regard to higher autonomic activation and larger pupil diameters. Interaction effects suggest that the presence of music influenced SloMo videos more strongly in perceived valence and arousal compared to real-time excerpts. Thus, while music enhanced both perceived and induced bodily arousal, stretched time in SloMo media does not simply induce heightened emotional states in observers, but rather affects them in cognitive dimensions related to valence and time perception.

### Time distortions

Perceiving distortions of time is ubiquitous and not limited to live-threatening situations [6] or pathological states as described by Oliver Sacks [48]. Previous research highlighted the lengthening of perceived duration for emotional stimuli that are more frequently experienced in daily life. For instance, viewing emotional pictures appears to last longer as compared to neutral ones [49], which is presumably related to increased arousal and emotional content (e.g., disgust versus fear). In the current study, we assumed arousal to be a crucial factor in participants’ responses and experiences of time. Using a holistic approach, we measured both cognitively perceived arousal as well as bodily reactions in terms of autonomic physiological activation and pupil diameter. Real-time video excerpts were perceived to be higher in arousal than SloMo excerpts. In addition, normalised deviations from clock-time duration were significantly different for SloMo excerpts, such that participants perceived them to be shorter than they actually were. Duration estimates for real-time excerpts matched clock-time duration more closely or were even judged to last longer. It should be noted that real-time videos were presented three times in a row in order to match the duration of the original music, so there might have been an advantage in judging the duration of the first real-time video. Nevertheless, the perceived deviations from clock-time are in line with previous research into arousal effects, since real-time videos also induced higher levels of bodily arousal in terms of respiration rate and larger pupil diameters. SloMo videos, in contrast, were both lower in perceived and felt arousal and underestimated in duration.

According to Arstila [7], increased arousal in situations of danger should lead to faster cognitive functioning, including heightened awareness of the surroundings and higher processing speed. When comparing the higher amount of information with an internal sense of normal speed, outer time is then interpreted as passing more slowly in these situations. Given the importance of arousal in the model of faster cognitive processing, the biological functionality of heightened states is plausible for moments of danger. On the other hand, less seems to be known about whether people occasionally experience pleasant, but emotionally intense, situations in SloMo. Although arousal may potentially explain the faster speed of cognitive processing even for a positive situation, there is typically no threat involved nor any need to respond quickly. There is some evidence that decelerating time is a useful mechanism in situations beyond immediate threat and danger. A recent study suggests that altered time perception can be a result of extensive training, in that expert athletes are able to make better predictions based on enhanced processing of simulated actions, which internally seem to be slowed down in time [50].

SloMo in films often appear to simulate such experiences for viewers [37]. Based on the findings of the current study, we argue that viewers may recognise that something crucial is happening in a film scene when SloMo is employed, and may emphasise with the characters more strongly. In a related paper, we found that there were more eye movements in SloMo film scenes, including fixation frequency as well as number of saccades and blinks [51]. So there is reason to argue that SloMo affects viewers in their attentional gaze behaviour. In the current study, judgments of perceived valence were also higher in SloMo, indicating that viewers did indeed perceive these scenes differently. Nevertheless, they do not necessarily respond to SloMo as if they were themselves in such highly emotional situations. Watching strong emotions on screen may thus not correspond with experiencing them veridically (cf. [38). This may be why film directors typically employ musical underscoring to increase arousal.

### The mediating effects of music

The accompanying music strongly affected arousal in the present study. Across all video genres, music as compared to no music induced higher arousal in terms of heart rate, respiration rate and skin conductance, and caused larger pupil diameters in viewers. Correspondingly, perceived arousal as well as valence were judged to be higher in conditions with music. These findings support previous research on music’s influence on perceived valence and arousal [21], as well as physiological activation and responses of the eye [16], [30]. The combination of these measures extends previous research, especially with regard to time perception.

We found that the presence of music not only increased arousal, but also enhanced the accuracy of duration estimates. Music may thus function as a pacemaker that facilitates timing judgements. Previous research occasionally manipulated the tempo of music and found that digitally slowed-down music reduced perceived duration, while accelerated music let to overestimations of time, possibly influenced by higher arousal [10], [11]. As mentioned before, the durations of the SloMo videos of our study were also underestimated, indicating that there could be cross-modal effects of stretched time both for visual and auditory stimuli. In another recent study, the tempo of the music was both accelerated and decelerated [52]. While the slower tempi in the digitally stretched music condition led to decreases in mean heart rate, no effects were found for versions in higher tempi. The presence of music on the whole caused higher heart rates as compared to silence.

Apart from arousal, music as a temporal art that works simultaneously on different time levels [8], [53], [54] may shape SloMo experiences to a crucial extent. Listeners may concentrate on a surface level to the actual sound events or notes, or perceive larger structures of bars and phrases. It may well be that focussing on such lower levels affects time perception and duration judgments to a significant extent, which could further modulate cross-modal SloMo experiences. Moreover, digital techniques in music production such as granular synthesis or brassage (both referring to sound fragmentation and re-composition at different speeds), in addition to the widely employed deceleration of tempi as used in some of the studies cited above, offer new ways of mediating time using sounds and music.

### Mediated motion in documentaries and films

SloMo is often thought to represent reality in a temporal close-up (such as in the German term “Zeitlupe”, lit. a magnifying time lens). In this sense, SloMo should make something visible that is inherently true. Media researchers, on the other hand, stress that slowing down the pace of the video playback can lead to a completely different perception of reality. As an example, Bernstein [55] demonstrates that court jurist’s judgments were distorted by a presentation of slowed-down footage of white police officers beating a male Afro-American motorist (Rodney King) after having stopped him for traffic violation. This example caused a wide debate on the truthfulness of media as evidence [39], because the act of police violence was visually perceived to be less harmful, and the role of the beaten man more active, in SloMo.

While SloMo may distort reality in documentary material, in films it is often employed to shift the viewer’s attention and to convey emotional meaning. The current study included a SloMo film scene from Clockwork Orange [40] that shows the main character attacking his friends with a metal chain. The accompanying classical music by Rossini, in contrast, suggests a positive valence of the scene, using a technique that has been called “contrapuntal music” in films [56]. In combination with SloMo, the viewer may perceive this scene to be more playful than the actual violence would suggest otherwise.

We found a number of differences relating to video genre. Films and sports excerpts were perceived to be higher in arousal compared to ballet, while ballet was more positive in valence. These results point to differences in the content and pace of the material, but may also indicate that viewers distinguish between documentary media and film drama. While there were no main effects of video genre on peripheral physiological responses, ballet scenes caused the largest pupil diameters. These effects were likely caused by the lower-level differences in the visual material, which could be more controlled in future research into SloMo effects.

### Limitations and future research

The current study presented pre-existing SloMo scenes from different sources, including commercial films and professional video clips of sport and ballet, all underscored with emotional music. The use of existing material typically has a downside in terms of varying stimulus lengths and differences in factors such as the music used or the luminance of the video pixels that may affect pupil size and viewers’ gaze direction. In order to compare SloMo and real-time scenes, we decided to present the real-time versions three times in succession so that the music was played in full duration. Participants were asked to judge the duration only of the first real-time presentation. While the two repetitions may have increased judgment accuracy as stated above, within both real-time conditions participants were still more accurate in the AV than in the V condition, indicating time distortions even for the real-time excerpts caused by the factor music. If the short real-time versions had been presented only once, this would have shortened the music and the time course of physiological reactions. Follow-up studies comparing different paces could use purpose-composed music that includes repetitions at a rate linked to the acceleration factors, or music could be digitally sped-up.

SloMo scenes are typically experienced relative to real-time footage in films, and slow passages or pieces in music such as symphony movements are interspersed and played in combination with faster ones. So the context of a SloMo film scene may crucially shape the viewer’s perception of relative time. Albeit we obtained a number of significant differences by contrasting SloMo and real-time versions, the interpretation and emotional meaning of SloMo scenes should be even stronger when presented in their context. By using longer segments in studies that include a range of playback speeds, time series of the different measures could be further analysed. Even more, recent innovations in virtual reality could be employed in order to simulate SloMo experiences in extreme situations as described above [6]. The presence of music may also modulate und influence duration estimation in these situations.

### Conclusions

Our results provide new insights into the impact of stretched time on perception and emotion. SloMo videos from film, sports and dance genres were perceived to be higher in valence but lower in arousal. Viewers’ pupillary diameters and respiration rates were reduced in SloMo. Music, on the other hand, increased perceived and induced arousal, particularly in SloMo conditions, and facilitated more accurate duration estimates.

Arousal is a key mechanism in psychological time perception, as corroborated by the results of this study. The higher arousal in life-threatening situations, on the other hand, leading to faster cognitive processing and distorted, slower time perception [7], is not similarly experienced by viewers of SloMo media. SloMo videos in the current study even caused psychophysiological relaxation and led to underestimations of time, perhaps because viewers internally related the decelerated movements to realistic movements in the real world. Thus SloMo in media may primarily change perceived valence and cognitive dimensions of duration estimates. Real-life experiences of distorted time are not necessarily related to slowing down the playback speed in media, albeit viewers may emphasise with the situations and individuals shown at such speed rates.

A number of open questions may inspire further research. The underlying psychological functions and processes of SloMo experiences in positive situations should be investigated. It can be speculated that such findings may explain the higher valence found for the SloMo excerpts in this study, or the distorted perception of documentary material [39], [55]. Furthermore, cross-modal effects on time perception should be investigated in relation to music’s impact on emotional arousal.
